# Adefovir dipivoxil-induced Fanconi syndrome associated with hypophosphatemic osteomalacia misdiagnosed as cervical spondylotic myelopathy: A case report and literature review

**DOI:** 10.1097/MD.0000000000046747

**Published:** 2025-12-19

**Authors:** Ke-jun Zhu, Long Xiang, Zhu-liu Chen, Jian-song Ji

**Affiliations:** a Graduate Joint Training Base of Zhejiang Chinese Medical University (Lishui Joint Training Base - Lishui Municipal Central Hospital), Lishui, China; b Department of Orthopedics, Qingyuan County People’s Hospital, Lishui, China.

**Keywords:** adefovir dipivoxil, cervical spondylotic myelopathy, Fanconi syndrome, hypophosphatemia, osteomalacia

## Abstract

**Rationale:**

Adefovir dipivoxil (ADV)-induced Fanconi syndrome associated with hypophosphatemic osteomalacia is an extremely rare disease. Owing to its rarity, it is easily misdiagnosed as cervical spondylotic myelopathy. Here, we present the case of a patient with Fanconi’s syndrome associated with hypophosphatemic osteomalacia that was misdiagnosed as having cervical spondylotic myelopathy.

**Patient concerns:**

A 44-year-old Chinese man with a 10-year history of ADV therapy for chronic hepatitis B presented with progressive bilateral lower extremity weakness for more than 6 months. Cervical magnetic resonance imaging revealed disc herniations at C4/5, C5/6, and C6/7, accompanied by spinal cord compression. Laboratory evaluations revealed glucosuria, proteinuria, hypophosphatemia, and impaired renal function. He was misdiagnosed with cervical spondylotic myelopathy and underwent anterior cervical corpectomy decompression and fusion. However, his lower limb weakness failed to improve 10 months after surgery.

**Diagnosis:**

On the basis of the patient’s clinical symptoms, along with his long history of oral ADV use, hypophosphatemia, and renal insufficiency, he was diagnosed with adefovir dipivoxil-induced Fanconi syndrome.

**Interventions:**

ADV was replaced with entecavir, and the patient was prescribed oral calcitriol 0.25 μg daily and oral calcium carbonate with vitamin D₃ (300 mg calcium and 60 IU vitamin D₃ per tablet) once daily.

**Outcomes:**

The patient’s lower limb muscle strength returned to normal after 6 months.

**Lessons:**

Fanconi syndrome with hypophosphatemic osteomalacia induced by low-dose adefovir dipivoxil (10 mg/d) is extremely rare. The condition may present with pain, muscle weakness, or numbness, and its nonspecific manifestations can easily lead to a misdiagnosis of cervical spondylotic myelopathy. For patients receiving long-term ADV therapy who develop such symptoms, especially in the presence of cervical disc herniation with spinal cord compression, clinicians should carefully evaluate laboratory findings and physical examination results to exclude the possibility of ADV-induced Fanconi syndrome.

## 1. Introduction

Hypophosphatemia osteomalacia is a metabolic bone disease characterized by low blood phosphorus levels, high urinary phosphorus levels, and low 25(OH)D levels. The characteristic clinical features of this disease include progressive bone pain, muscle weakness, difficulty in walking, and fracture. Hypophosphatemia osteomalacia is most often hereditary or cancer-related. Several case reports have associated adefovir dipivoxil (ADV), an antiviral agent commonly used to treat hepatitis B, with hypophosphatemia osteomalacia.^[1–3]^ We report the case of a 44-year-old man who underwent cervical discectomy for cervical disc herniation but was subsequently diagnosed with adefovir dipivoxil-induced Fanconi syndrome associated with hypophosphatemic osteomalacia.

## 2. Case presentation

A 44-year-old man presented with a 6-month history of bilateral lower limb numbness and progressive gait disturbance. He had a 10-year history of chronic hepatitis B and had been receiving adefovir dipivoxil (10 mg daily) for 6 years. He also had a history of chronic renal insufficiency and was initially evaluated at a local hospital.

On physical examination, Hoffmann’s sign was negative bilaterally, and muscle strength in the upper limbs was normal without increased tone. The straight leg raising test was negative. Superficial sensation was diminished below the iliac and inguinal regions bilaterally. Muscle strength was approximately grade III in the left lower limb and grade I in the right lower limb, with increased tone in both legs. Bilateral knee and Achilles tendon reflexes were brisk (+++). Babinski’s sign was negative on the right but suspected to be positive on the left.

**Figure 1.**
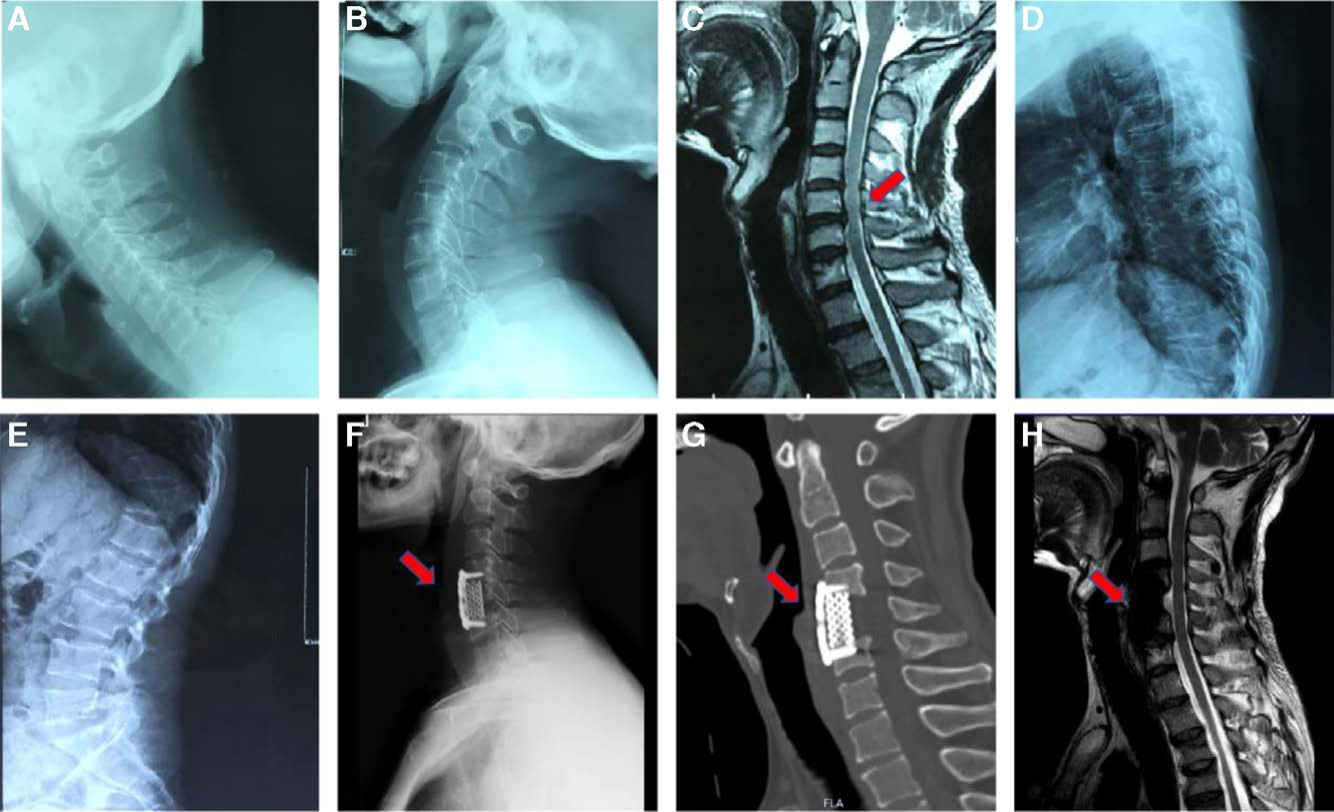
Pre- and postoperative imaging findings of a 44-year-old man who underwent cervical discectomy for cervical disc herniation and was later identified as having adefovir dipivoxil-induced Fanconi syndrome associated with hypophosphatemic osteomalacia. (A, B) Preoperative lateral radiographs of the cervical spine in extension and flexion positions. (C) Sagittal MR image showing disc herniations at C4/5, C5/6, and C6/7 (red arrow), with corresponding spinal cord compression. (D and E) Lateral radiographs of the thoracic and lumbar spine demonstrating multiple flattened vertebral bodies with biconcave changes, suggesting osteomalacia. (F–H) Postoperative follow-up images. (F) Lateral cervical radiograph, (G) CT scan, and (H) sagittal MR image confirming stable internal fixation (red arrows) and satisfactory spinal cord decompression.

Laboratory tests from the local hospital revealed routine urine tests indicating the presence of 2 + glucose. Blood biochemistry revealed an alkaline phosphatase concentration of 232 U/L, a creatinine concentration of 10.6 μmol/L, a uric acid concentration of 7.7 μmol/L, and a blood phosphorus concentration of 0.6 mmol/L. Lateral radiographs of the thoracic and lumbar spine revealed multiple flattened vertebral bodies with biconcave changes (Fig. 1D and E). Magnetic resonance imaging revealed disc herniations at C4/5, C5/6, and C6/7 (Fig. 1C), corresponding to spinal cord compression and a diagnosis of cervical myelopathy. The patient underwent anterior cervical corpectomy and decompression with fusion at the local hospital. The procedure included discectomy at the C4/5 and C5/6 levels and partial resection of the C5 vertebral body. (Fig. 1F–H).

The patient initially showed partial improvement in lower limb muscle strength after surgery and was discharged for continued rehabilitation. However, despite rehabilitation, he remained unable to walk, and 10 months later, he was admitted to our hospital for further evaluation.

On examination, hand grip strength was normal, and Hoffmann’s sign was negative bilaterally. The upper limb muscle strength was approximately grade IV, whereas the lower limb muscle strength was approximately grade III. Superficial sensation was mildly decreased below the iliac and inguinal regions bilaterally. Muscle tone was normal, bilateral knee reflexes were moderately brisk (++), and Babinski’s sign was suspected to be positive on the left side.

Upon admission, comprehensive tests were conducted. Routine blood tests, C-reactive protein levels, coagulation function, tumour marker levels, and thyroid function were normal. The sedimentation rate was 4.0 mm/h. Routine urine tests revealed 2 + glucose. Blood biochemistry revealed an alkaline phosphatase concentration of 225 U/L, a creatinine concentration of 112 μmol/L, and a uric acid concentration of 89 μmol/L (Table 1). Blood potassium, sodium, calcium, and magnesium levels were normal, and the blood phosphorus concentration was 0.38 mmol/L. The serum 25(OH)D concentration was 25.0 ng/mL, the β-bone collagen cross-linking concentration was 645 pg/mL, the propeptide of type I procollagen concentration was 169.80 ng/mL, and the osteocalcin concentration was 31.76 ng/mL. Electromyography revealed no evidence of peripheral nerve damage. Cranial magnetic resonance imaging revealed no obvious abnormalities. The bone mineral density indicated osteoporosis.

**Table 1:** Laboratory results at first admission, second admission and 6-month follow-up.

	First admission (local hospital)	Second admission (our hospital)	Last follow-up (our hospital)	Reference
Sedimentation rate (mm/h)	5.2	4.0	3.5	0.0–15.0
Urine glucose (mmol/L)	++	++	NA	–
ALP (U/L)	232	225	110	45–125
Cr (μmol/L)	106	112	105	57–97
Uric acid (μmol/L)	77	89.	139	90–420
K (mmol/L)	3.81	3.64	4.30	3.5–5.3
Na (mmol/L)	139.2	138	137.9	137–147
Cl (mmol/L)	108.9	112.3	104.1	99–110
Ca (mmol/L)	2.23	2.19	2.20	2.11–2.52
Mg (mmol/L)	0.94	0.98	0.85	0.75–1.02
P (mmol/L)	0.6	0.38	0.95	0.85–1.51
PCT (pg/mL)	NA	5.69	6.45	<9.52
25(OH)D (ng/mL)	NA	25.00	27.12	>20.00
β-CTX (pg/mL)	NA	644.5	508.95	43–783
P1NP (ng/mL)	NA	169.80	66.51	9.06–76.24
Osteocalcin (ng/mL)	NA	31.76	25.34	6.00–24.66

25(OH)D = 25-hydroxyvitamin D, ALP = alkaline phosphatase, Cr = creatinine, PCT = procalcitonin, PINP = procollagen type I N-terminal propeptide, β-CTX = beta-cross-linked C-telopeptide of type I collagen.

After discussion by a multidisciplinary panel, including infectious disease and endocrinology specialists, the diagnosis was made as Fanconi syndrome associated with adefovir dipivoxil combined with hypophosphatemia. Adefovir dipivoxil was discontinued and replaced with entecavir. In addition, the patient was treated with oral calcitriol 0.25 μg once daily and oral calcium carbonate with vitamin D₃ (equivalent to 300 mg of elemental calcium and 60 IU of vitamin D₃ per tablet; Caltrate D, Pfizer Consumer Healthcare), one tablet daily. Following this comprehensive management, the patient’s clinical symptoms improved. At the 6-month follow-up, the patient’s lower extremity muscle strength had fully recovered, and the serum phosphorus concentration had returned to 0.95 mmol/L (Table 1).

## 3. Timeline

**Figure 2.**
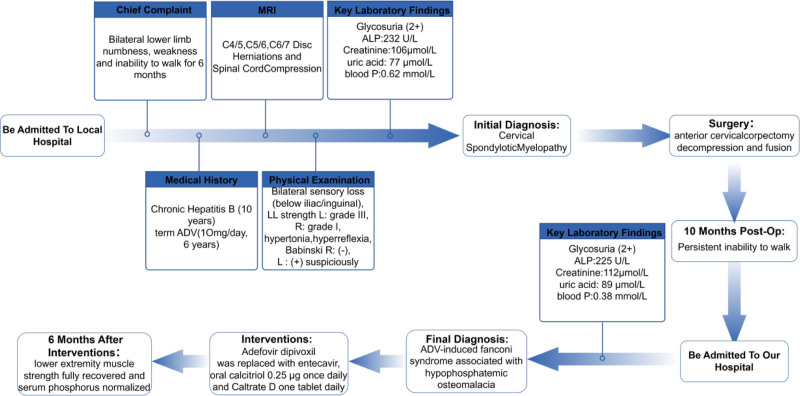
Patient treatment course. ADV = adefovir dipivoxil, ALP = alkaline phosphatase.

The patient’s treatment course is illustrated in Figure 2.

## 4. Patient perspective

At first, I was really frustrated because I couldn’t walk. After the surgery, when I started feeling some strength returning in my legs, I felt hopeful, but I was also worried that the surgery might not work. Ten months later, my leg strength still hadn’t fully recovered, and I felt depressed about it. During the 6-month course of treatment, I often felt anxious. However, after completing the final round of treatment, my leg strength finally came back, and my quality of life improved. I’m really happy with the outcome.

## 5. Informed consent

Written informed consent was obtained from the patient for the publication of this study.

## 6. Discussion

ADV is a commonly used antiviral agent for the treatment of chronic hepatitis B. While renal toxicity typically occurs at doses of 30 mg/d, it can also occur at lower doses, such as 10 mg/d, with dose- and time-dependent effects.^[4–6]^ The mechanism of ADV-induced renal toxicity involves binding to anion transporter-1 in the renal tubule epithelium, where it inhibits mitochondrial DNA polymerase. This results in vacuolar degeneration, apoptosis, and necrosis of renal tubular epithelial cells.^[5]^

Fanconi syndrome, often accompanied by hypophosphatemic osteomalacia, is a well-documented complication of long-term, low-dose adefovir dipivoxil therapy.^[7]^ The most common symptom of this syndrome is bone pain.^[2]^ In our case, the patient developed lower limb numbness and difficulty walking after 6 months of oral ADV therapy. The patient was initially diagnosed with cervical spondylotic myelopathy and underwent discectomy, but symptoms persisted. Upon further examination, laboratory tests revealed increased urine sugar and protein levels, decreased blood phosphorus levels, abnormal renal function, and bone scintigraphy, suggesting osteomalacia. These findings led to a diagnosis of Fanconi syndrome associated with hypophosphatemic osteomalacia. After discontinuing ADV, the patient’s symptoms, including numbness and muscle strength, significantly improved.

Clinicians should be vigilant in considering adverse drug reactions when patients on long-term ADV therapy present with unexplained pain, mobility issues, muscle weakness, or numbness. In addition to routine renal function monitoring, attention to blood phosphorus and urine changes is crucial for the early detection of renal damage. In cases where patients exhibit systemic pain, fatigue, numbness, and abnormal renal function, further evaluation for secondary Fanconi syndrome is necessary. Once diagnosed, discontinuing ADV is critical to prevent further complications.

Osteomalacia and cervical spondylotic myelopathy can present with similar clinical features, such as motor dysfunction, muscle weakness, and sensory disturbances. These overlapping symptoms can lead to diagnostic confusion, particularly in the early stages. This is further complicated by the fact that some spine surgeons may not be fully aware of the toxic effects of hepatitis B medications, which can result in misdiagnosis. Zhao et al^[8]^ reported that muscle weakness and fatigue were present in 100% of patients with osteomalacia, with bone pain primarily in the heel, lower back, and ribs. However, owing to the nonspecific nature of these symptoms, a significant portion of patients (33.6%, 47 of 140 patients) were misdiagnosed, including some who were mistakenly diagnosed with spondyloarthropathy.

To avoid misdiagnosis in similar cases, clinicians should be aware of early warning signs, such as abnormal blood phosphorus levels, renal function abnormalities, and urinary changes.^[9]^ Notably, serum phosphate levels in patients with ADV-induced Fanconi syndrome may be only modestly low, with values reported between 0.3 and 0.7 mmol/L in prior case series.^[10]^ As the serum phosphate level is a poor indicator of the concentration of total body phosphate, which is primarily stored intracellularly, careful attention to these factors is crucial. Additionally, imaging findings in Fanconi syndrome can be nonspecific, often resembling those of osteoporosis, which is common in patients with spinal diseases. As a result, spine surgeons may overlook radiographic signs of osteomalacia, such as cortical thinning, increased medullary lucency, blurred or absent trabeculae, and vertebral body deformities such as biconcave shapes, kyphosis, and scoliosis.

In some cases, bone densitometry and radioisotope scans may help differentiate osteomalacia from osteoporosis.^[11]^ Bone biopsy with double tetracycline labeling remains the gold standard for diagnosing osteomalacia and distinguishing it from osteoporosis.^[12]^ During physical examination, clinicians should be cautious when myelopathic signs are present or suspected, even if imaging suggests nerve compression. Some patients with normal findings may still exhibit myelopathic signs, leading to potential misinterpretation of conditions such as osteomalacia.^[13]^

Given this, it is imperative for clinicians to appreciate the vital importance of interdisciplinary collaboration, as the underlying causes of common clinical symptoms are often complex. For instance, the evaluation of patients presenting with atypical pain mandates a comprehensive assessment that incorporates trauma history, medication use, and neurological factors. However, psychological comorbidities are often overlooked if psychiatrists are not involved in the patient’s treatment process, which can hinder the implementation of effective treatment strategies.^[14]^ Therefore, treatment strategies should also consider multiple perspectives. Recent research has highlighted that vagus nerve stimulation can play a critical role in treating cervicogenic headaches that are refractory to conventional therapies, demonstrating the potential benefits of multidisciplinary treatment for patients.^[15]^ In this case, the patient initially sought consultation in the spinal surgery department. Through collaboration with spinal surgeons, infectious disease specialists, and endocrinologists, the diagnosis was confirmed, and effective interventions were implemented, further underscoring the paramount importance of interdisciplinary collaboration in the management of rare and complex conditions.

In conclusion, for patients on long-term ADV therapy, particularly elderly patients, monitoring not only renal function but also changes in blood phosphorus and urine is essential. Early detection of kidney damage and timely diagnosis of Fanconi syndrome can significantly improve patient outcomes. Clinicians should consider the full clinical picture, including patient history, physical examination, and laboratory results, to avoid misdiagnosis and ensure appropriate management.

## 7. Conclusion

We present the case of a 44-year-old man who developed Fanconi’s syndrome associated with hypophosphatemic osteomalacia caused by low-dose ADV therapy (10 mg/d) and who was misdiagnosed with cervical spondylotic myelopathy. Aetiological treatment is the most effective treatment for adefovir dipivoxil-induced Fanconi syndrome.

## Author contributions

**Data curation:** Zhu-liu Chen.

**Investigation:** Long Xiang.

**Project administration:** Jian-song Ji.

**Writing – review & editing:** Ke-jun Zhu.
